# A dataset for voice-based human identity recognition

**DOI:** 10.1016/j.dib.2022.108070

**Published:** 2022-03-18

**Authors:** Baha’ A. Alsaify, Hadeel S. Abu Arja, Baskal Y. Maayah, Masa M. Al-Taweel

**Affiliations:** Department of Network Engineering and Security, Jordan University of Science and Technology, P.O. Box 3030, Irbid 22110, Jordan

**Keywords:** FLAC, Same phrase, Audio dataset, Different phrase, Voice recognition, Applied machine learning

## Abstract

This paper introduces a new English speech dataset suitable for training and evaluating speaker recognition systems. Samples were obtained from non-native English speakers from the Arab region over the course of two months. The dataset was divided into two sub-datasets. Ten samples were collected from each speaker for each sub-dataset. The first sub-dataset contains samples of speakers repeating the phrase “Machine learning 1, 2, 3, 4, 5, 6, 7, 8, 9, 10”. The second sub-dataset contains samples for the same speakers speaking randomly for five to ten seconds for each sample. The dataset consists of 150 speakers with a total of 3,000 data samples and about six hours of speech.

## Specifications Table

**Table:** 

Subject	Computer Science, Applied Machine Learning
Specific subject area	Speaker identification
Type of data	Audio Table
How data were acquired	Voice samples were recorded using different recording hardware (e,g. microphones, mobile devices, and laptops) then uploaded and stored on cloud applications. Data samples were collected over the course of two months in real-world, noisy environments.
Data format	Raw data
Description of data collection	The data collection process was divided into two groups. Each group comprised of ten samples from each speaker. For the first group, speakers repeat the same phrase ten times. For the second group, speakers speak different phrases. The length of each phrase is approximately ten seconds.
Data source location	Institution: Jordan University of Science and Technology, Network Engineering and Security Department. City/Town/Region: Irbid Country: Jordan
Data accessibility	Repository name: Mendeley Data Identification number: 10.17632/zw4p4p7sdh.1 Direct URL to data: https://data.mendeley.com/datasets/zw4p4p7sdh/1

## Value of the Data

• Many existing datasets [1] are obtained under controlled conditions. This dataset was obtained in an uncontrolled, noisy environment.
• This dataset is beneficial for organizations and individuals working in the machine/deep learning field to develop voice-based products and services.
• This dataset is raw voice data that did not undergo any filtering process, which leaves the absolute choice for developers to pass it through their choice of processing procedure.
• This dataset provides insights on the accents spoken by English speakers of Middle Eastern descent.
• With height and weight provided as extra information about the speaker, studies can be done on the relationship between a speaker's utterances and their physical attributes.

## 1 Data Description

This dataset is divided into two main sub-datasets: samePhrase and differentPhrase. Each speaker has the same label in both sub-datasets. In the samePhrase sub-dataset, a speaker repeats the sentence “Machine Learning 1, 2, 3, 4, 5, 6, 7, 8, 9, 10” ten times. The length of each sample is between seven and ten seconds. For the differentPhrase sub-dataset, each speaker contributed with a phrase selected randomly from different resources such as books, song's lyrics, or online texts. Each speaker contributed with ten different samples, the length of each sample in the differentPhrase sub-dataset does not exceed ten seconds. Table 1 shows in detail the division of the dataset.

**Table 1:** Dataset subsets.

		Number of Speakers		
Subsets	Number of Samples	Female	Male	Data Duration
samePhrase	1500	96	54	2.9 h
differentPhrase	1500	96	54	3.1 h
**TOTAL**	3000	150		6 h

**Fig. 1:**
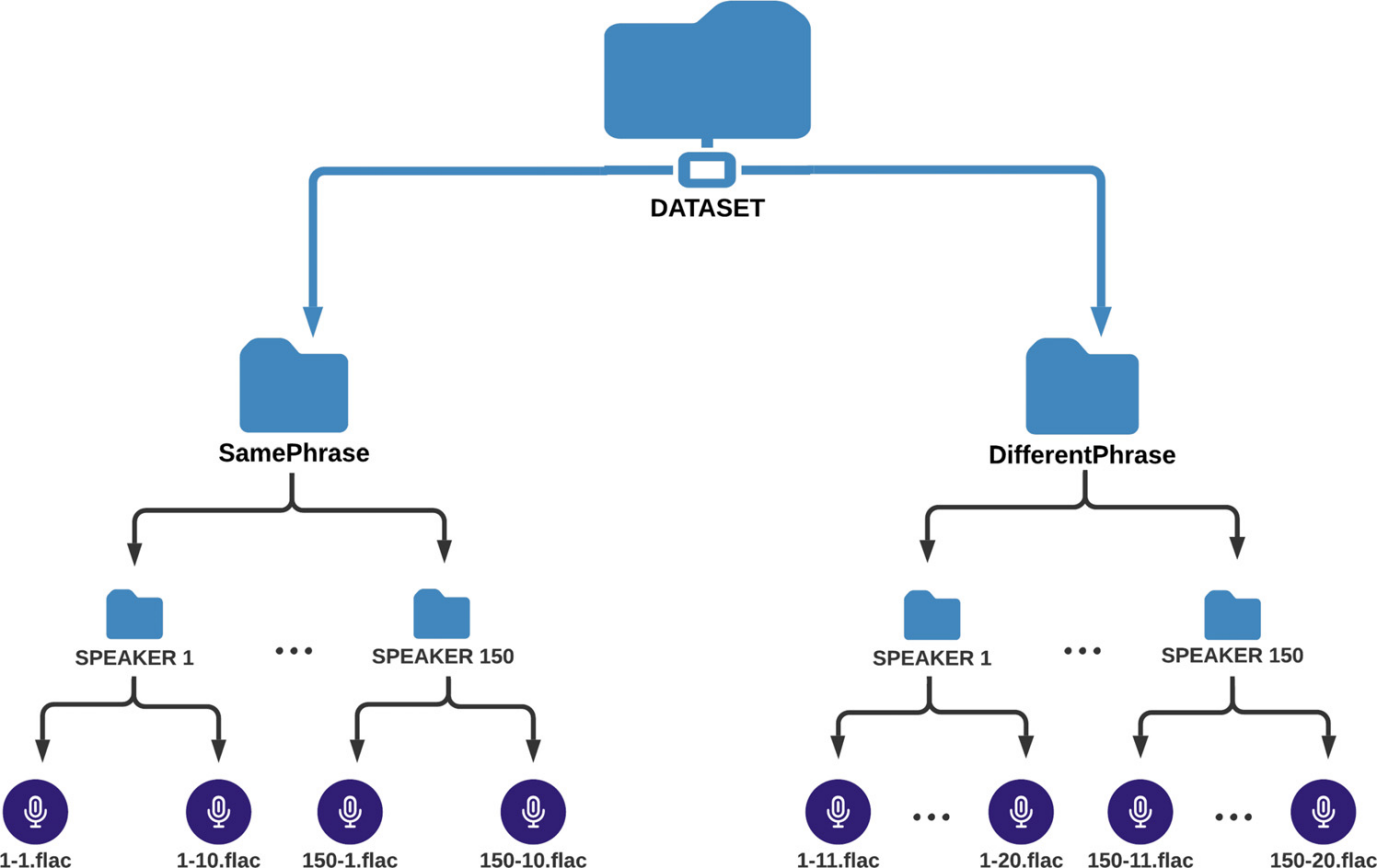
Architecture of the dataset.

In both the samePhrase and differentPhrase sub-datasets, there are 150 directories labeled from 1 to 150 that corresponds to the speaker who contributed to the dataset. Each directory holds ten voice samples for each speaker. The naming convention for each sample follows “Speaker Label - Sample Number” format. For example, “3–4.flac” refers to the 4th voice sample provided by the 3rd speaker. For the samePhrase sub-dataset, the samples are numbered from one to ten for each speaker. While for the differentPhrase sub-dataset, the samples are numbered from 11 to 20 for each speaker. The directories hierarchy for the dataset and the samples numbering scheme are provided in Fig. 1.

## 2 Experimental Design, Materials and Methods

### 2.1 Subjects

In this study, a total of 150 healthy speakers (96 females, and 54 males) voluntarily participated in the data collection process. The speakers had a mean ± standard deviation age, height, and weight values of 22.13 ± 6.56 years, 163.4 ± 20.6 cm, and 66.12 ± 20.3 kgs, respectively. More details regarding the speakers’ statistics can be found in Table 2. Before recording their voices, the experiment parameters were explained to the participating speakers. The details for the individual participating speakers can be found in the speakers.csv file [2], which is uploaded together with the dataset files.

**Table 2:** Speakers physical statistics.

	Male				Female			
	Min	Max	Mean	Standard Deviation	Min	Max	Mean	Standard Deviation
**Age**	9	61	22.51852	8.211223	8	52	22.12371	5.868805
**Weight**	32	177	76.7037	20.35128	20	166	60.42268	18.66248
**Height**	134	190	174.8889	9.77418	132	177	161.4021	6.366677

### 2.2 Experimental procedure

Each speaker participating in the data collection process was asked to record 20 voice samples. Out of the 20 samples, ten samples represent the phrase “Machine Learning, 1, 2, 3, 4, 5, 6, 7, 8, 9, 10” which are included in the samePhrase sub-dataset. As for the remaining ten samples, the choice was left for the speaker to select the phrases they wish to say. These samples are included in the differentPhrase sub-dataset.

**Fig. 2:**
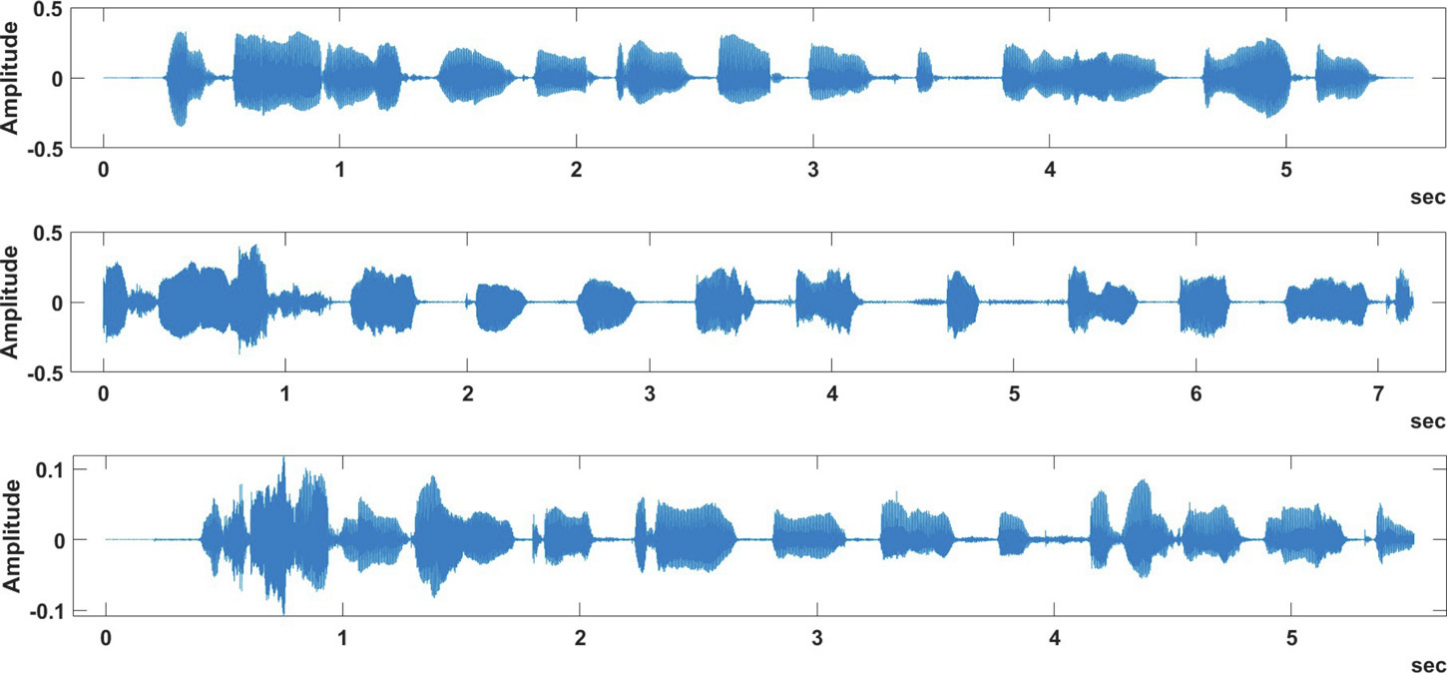
Different speakers saying same phrase in the samePhrase sub-dataset.

**Fig. 3:**
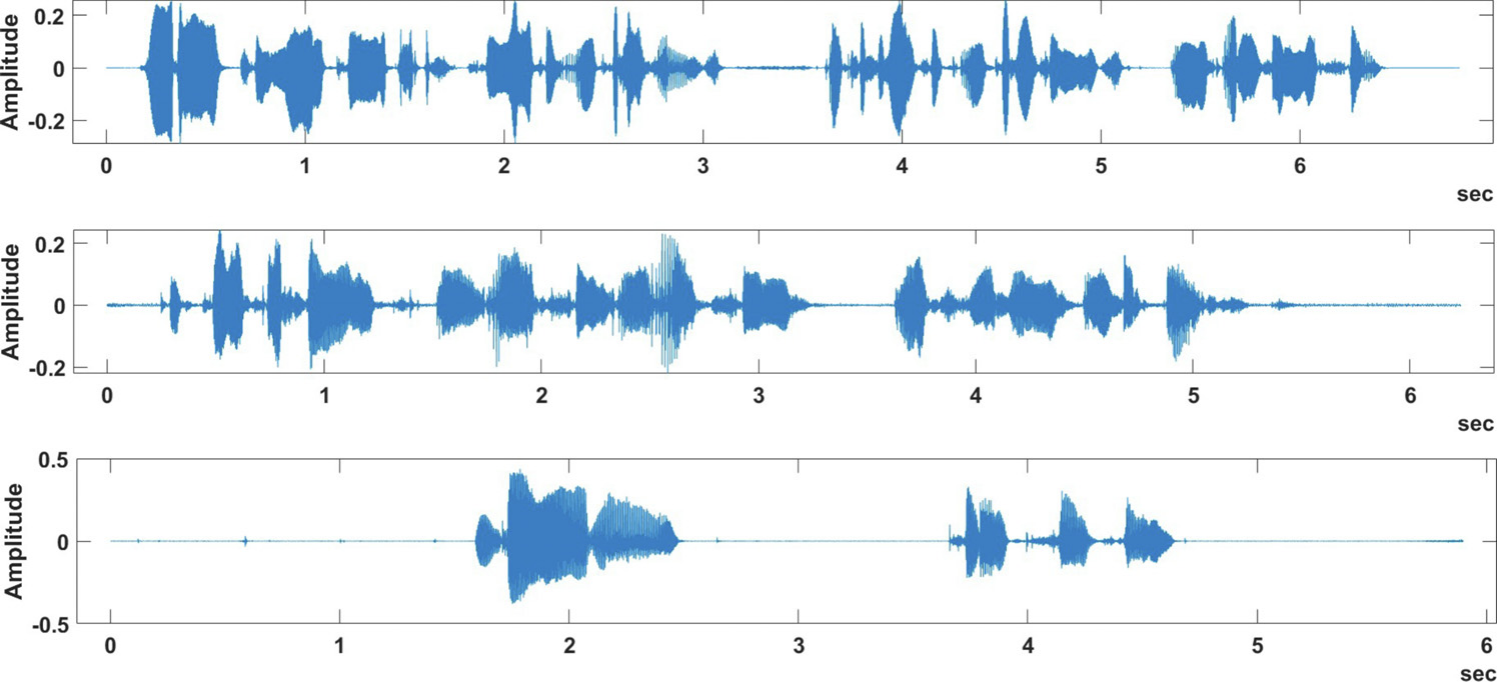
Different speakers saying different phrase in the differentPhrase sub-dataset.

**Fig. 4:**
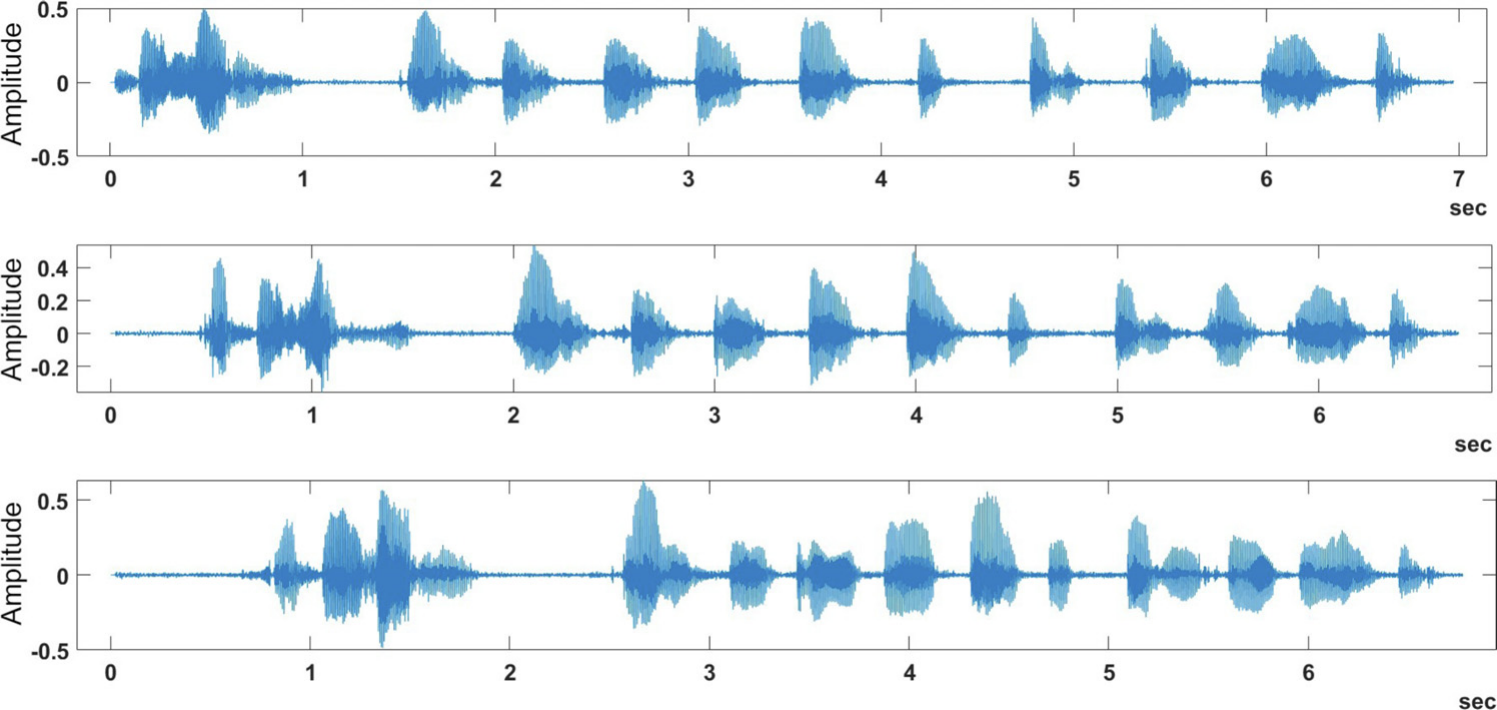
Same speaker saying same phrase in the samePhrase sub-dataset.

**Fig. 5:**
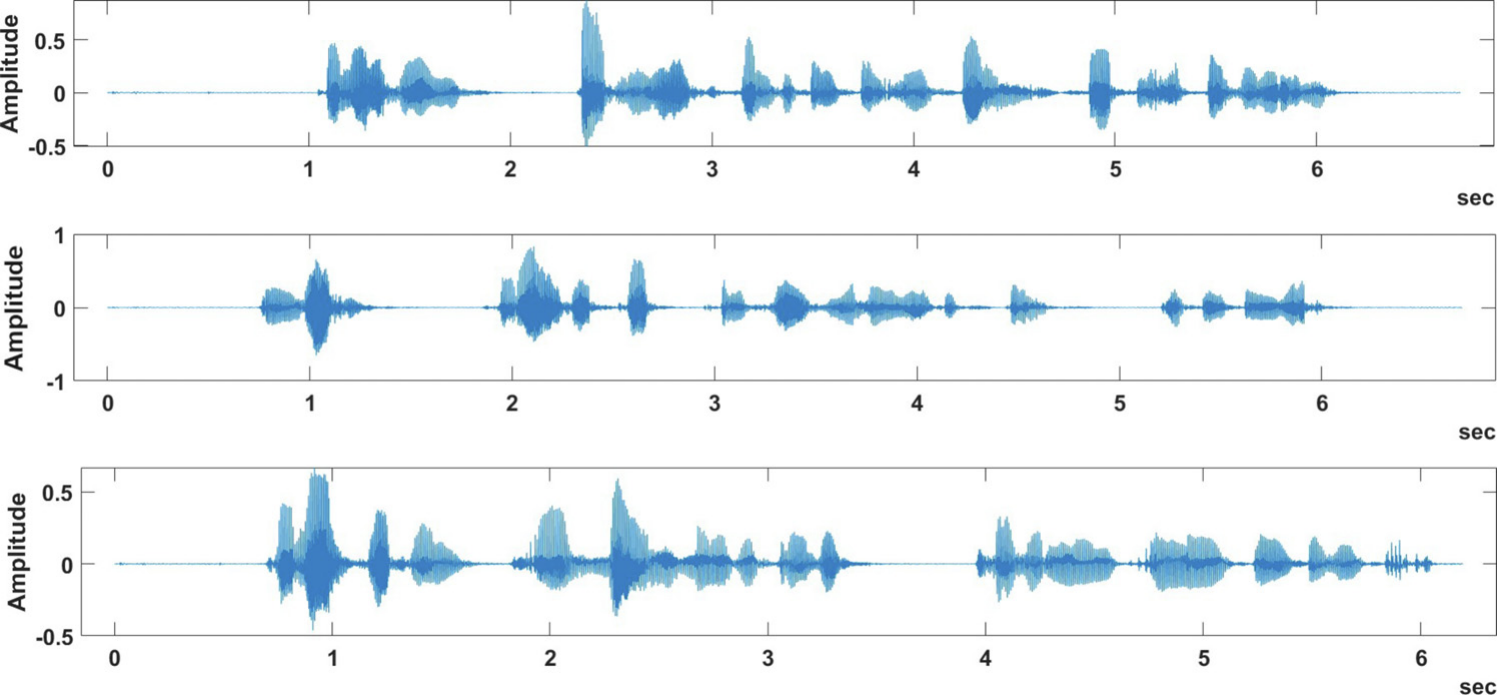
Same speaker saying different phrase in the differentPhrase sub-dataset.

Fig. 2 and Fig. 4 provide recording samples from the samePhrase sub-dataset, while Fig. 3 and Fig. 5 provide recording samples from the differentPhrase sub-dataset.

Fig. 2 provides three sample plots of the phrase “Machine Learning, 1, 2, 3, 4, 5, 6, 7, 8, 9, 10” spoken by three different speakers, while Fig. 4 shows the same phrase being spoken by the same speaker for three times. By examining these figures, we can easily view the variations in the same phrase whether it was spoken by the same speaker or by a different speaker. On the other hand, Fig. 3 shows different speakers saying different phrases, while Fig. 5 shows the same speaker saying different phrases. These variations in the spoken phrases can be utilized to build classification models to achieve many objectives such as speaker identification and speech recognition.

By examining Fig. 2, it is noted that there is a similarity between the same phrase plotting, even though it is by different speakers. Fig. 4 shows more similarity in the same phrase plot than in Fig. 2 due to the plots being generated by the same speaker, which is shown through the value of the amplitude that is more convergent than the previous plot.

### 2.3 Data collection

In this section, the different stages by which the collected data was checked, cleaned, and unified are described.

 **Stage1. Collecting Samples and labeling**
  In this stage, we gathered all the samples received from volunteering speakers who used different communication platforms (e.g., Messenger, WhatsApp, Cloud Apps, …) into one dataset. The collected samples were then divided into two sub-datasets based on the phrase.
 **Stage2. Filtering samples**
  All samples were checked manually, and each voice record was played to make sure that it was free of any overlapping speech noise. In other words, if we detected any other voice in the background that might affect the coherence of the phrase the speaker is saying, it will be considered a noise, and the voice sample will be dropped. For the samePhrase sub-dataset, we made sure that samples abide by the phrase “Machine Learning 1, 2, 3, 4, 5, 6, 7, 8, 9, 10”. Any speaker sent samples that did not abide by the instructions was removed from the dataset, and its label was dropped from the csv file that contains the speakers’ information.
 **Stage3. Renaming files**
  Each file was renamed to start with its speaker label, and then a dash, then the number of the sample. For example, if a speaker has label 149 then the first sample in the samePhrase sub-dataset will be “149–1.flac” while the last sample in the samePhrase sub-dataset will have the label “149–10.flac”. The same speaker will have entries in the differentPhrase sub-dataset that will start with label “149–11.flac” and will end with “149–20.flac”.
 **Stage4. Unifying the format**
  Samples collected were received in various formats. For example, samples collected via WhatsApp have the .ogg [3] extension while Facebook messenger supports the .mp4 format [4]. All the collected samples were converted to .flac [5] format to have a unified format.

## Ethics Statement

During the time of data collection, each speaker was provided with a consent form to sign which declares explicitly that personal information will not be disclosed, and that each participating speaker has the right to stop participating in the process at any time if they chose to do so.

## Author Contributions

Conceptualization, B. Alsaify; Methodology, B. Alsaify; Software, H. Abu Arja, B. Maayah,

M. Al-Taweel; Validation, B. Alsaify, H. Abu Arja, B. Maayah, M. Al-Taweel; Data Curation,

H. Abu Arja, B. Maayah, M. Al-Taweel; Writing - Original draft preparation, H. Abu Arja, B. Maayah, M. Al-Taweel; Writing - Review & Editing, B. Alsaify, H. Abu Arja, B. Maayah, M. Al-Taweel; Supervision, B. Alsaify.

## Declaration of Competing Interest

The authors declare that they have no known competing financial interests or personal relationships which have or could be perceived to have influenced the work reported in this article.

## Data Availability

A Dataset for Voice-Based Human Identity Recognition (Original data) (Mendeley Data).
